# The Incidence of Hypothyroidism Following Hemithyroidectomy in a Tertiary Academic Center in Saudi Arabia

**DOI:** 10.7759/cureus.35703

**Published:** 2023-03-02

**Authors:** Amani Alhozali, Abdulsalam Alqutub, Mohammad S Ahmed, Omar A Alsulami, Khaled Alfawaz, Hassan Faidah, Omar AlNoury, Nawaf Alquliti, Mazin Merdad

**Affiliations:** 1 Department of Internal Medicine, Faculty of Medicine, King Abdulaziz University, Jeddah, SAU; 2 Department of Medicine, Faculty of Medicine, King Abdulaziz University, Jeddah, SAU; 3 Department of Otolaryngology - Head and Neck Surgery, Faculty of Medicine, King Abdulaziz University, Jeddah, SAU

**Keywords:** retrospective study, risk factors, incidence, complications, hypothyroidism, hemithyroidectomy

## Abstract

Background

Hemithyroidectomy is a common procedure used to treat various benign and malignant conditions. It is often associated with complications, of which hypothyroidism is an underappreciated sequel. We sought to comprehend the rate and associated risk factors for developing hypothyroidism following hemithyroidectomy at King Abdulaziz University Hospital (KAUH).

Methods

In this retrospective study, we reviewed the medical records of all patients who had hemithyroidectomies for benign and malignant conditions between January 2008 and August 2022. Patients were analyzed for age, gender, body mass index (BMI), comorbidities, family history of thyroid disease, thyroid antibodies, and pre- and postoperative thyroid-stimulating hormone (TSH). Pre- and postoperative TSH levels were compared using the Wilcoxon signed-rank test.

Results

From 153 cases, 39 patients met the inclusion criteria; 31 (79.5%) were females. Seventeen (43.59%) patients developed biochemical hypothyroidism within two years following hemithyroidectomy; the majority (64.71%) of those with hypothyroidism developed it within the first six months. There was a significant increase in TSH levels following surgery (p < 0.001).

Conclusion

The overall incidence of hypothyroidism within two years of hemithyroidectomy is 43.59%; among those who developed hypothyroidism, the majority (64.71%) did so within the first six months. Thus, we strongly recommend continuous monitoring of TSH levels in the first six months, as it may aid in the decision to begin treatment before symptoms develop.

## Introduction

Thyroid lobectomy, also known as hemithyroidectomy, is a surgical procedure involving removing half of the thyroid gland with or without the isthmus while leaving the contralateral lobe intact [1]. Hemithyroidectomy treats various conditions, including benign and malignant thyroid diseases and pathologically indeterminate nodules [2,3]. Postoperative complications include surgical site infections, bleeding, and vocal cord paralysis [4]. Although hypothyroidism following hemithyroidectomy cannot be predicted with certainty, its detrimental impact on patients’ health cannot be overstated [5]. However, one thyroid lobe should theoretically contain enough thyrocytes to maintain normal thyroid function [6].

Many studies have described the association between hemithyroidectomy and hypothyroidism. According to a meta-analysis, the rates of developing hypothyroidism following hemithyroidectomy range from 0% to 43%, with a pooled risk of 22% [7,8]. Some reports showed that the prevalence of hypothyroidism after thyroid lobectomy (including hemithyroidectomy with isthmusectomy) ranges between 6.5% and 45% [9]. The lack of a standardized follow-up duration and definition of hypothyroidism contributes to the wide range of reported incidences [10]. Previous studies have shown that subclinical hypothyroidism or clinical hypothyroidism can occur after hemithyroidectomy, with subclinical hypothyroidism being much more common than clinical hypothyroidism [7,11].

However, the incidence and associated risk factors for developing hypothyroidism following hemithyroidectomy are not fully understood. Different hypothyroidism definitions, the timing of thyroid hormone detection, the duration of follow-up, studied populations, preoperative thyroid function status, and surgical techniques may all impact the results. Our primary aim is to estimate the incidence of postoperative hypothyroidism in patients who have undergone hemithyroidectomy at King Abdulaziz University Hospital (KAUH). Furthermore, the secondary aim of this study is to find the risk factors associated with the development of hypothyroidism following hemithyroidectomy in KAUH from 2008 to 2022.

## Materials and methods

In this retrospective study, we reviewed the records of all patients who were at least 18 years old and underwent hemithyroidectomy in KAUH between January 2008 and August 2022. The study was approved by the Institutional Review Board (IRB) of our hospital (reference number: 185-22). We excluded all patients who had previous neck radiation exposure, all those who were on antithyroid drugs, abnormal thyroid function tests prior to surgery, and patients with incomplete data, such as not having documented thyroid function tests in the first two years postoperatively. We obtained the medical record number, demographics such as age and gender, and comorbidities such as dyslipidemia, diabetes mellitus (DM), hypertension, and chronic kidney disease. Additionally, we obtained the smoking status, body mass index (BMI), and condition type, whether it is benign or malignant, which lobe was resected (right or left), if the isthmus was resected or not, and the presence of thyroid antibodies including antithyroid peroxidase (TPO) antibody and anti-thyroglobulin antibody, and pre- and postoperative thyroid-stimulating hormone (TSH). All patients with a postoperative TSH level > 4.4 mIU/L were labeled as having biochemical hypothyroidism. For descriptive statistics, continuous variables are presented as means ± standard deviation, and they were compared using the Student t-test. Categorical variables are summarized using number and frequency, and the chi-square and Pearson tests were used to compare them. For nonparametric data, the Mann-Whitney and Wilcoxon signed-rank tests were used. Data were entered into Google Forms (Google, Inc., Mountain View, CA, USA), then extracted into Excel version 16.0 (Microsoft Corp., Redmond, WA, USA), and analyzed using the Statistical Package for the Social Sciences for Windows version 21.0 (IBM SPSS Statistics, Armonk, NY, USA). A p-value of <0.05 was considered significant for all tests.

## Results

Overall, 153 patients underwent hemithyroidectomy in KAUH between 2008 and 2022, and 39 met the inclusion criteria. Seventy were excluded due to the lack of consistent follow-up, 25 patients were excluded due to missing data, and 19 patients had abnormal preoperative thyroid profiles. As shown in Table 1, most were females (n = 31, 79.5%), and the mean age at surgery was 40.67 ± 11.21. Four (10.3%) patients had a family history of thyroid disease. The majority of the patients (n = 27, 69.2%) had a benign condition as the indication for surgery; 23 (59%) had their right lobe resected, and 17 (43.59%) had an isthmusectomy alongside the lobectomy. Moreover, one (2.6%) patient had a positive anti-TPO antibody, two (5.1%) had a positive anti-thyroglobulin antibody, and three (7.7%) had both anti-TPO and anti-thyroglobulin antibodies positive, and the rest had negative antibodies. Surgeons from the otolaryngology department performed 21 (53.8%) surgeries, while the rest were done by general surgery. Table 1 shows the patients’ descriptive statistics.

**Table 1:** Patients’ demographics and characteristics

Variable	Number (%)
Gender	
Female	31 (79.5)
Male	8 (20.5)
Age at the time of surgery (years)	40.67 ± 11.21
Disease type	
Benign	27 (69.2)
Malignant	12 (30.8)
Thyroid antibody present	
Antithyroid peroxidase antibody positive	1 (2.6)
Anti-thyroglobulin antibody positive	2 (5.1)
Both antibodies positive	3 (7.7)
Both antibodies negative	33 (84.6)
Family history of thyroid disease	
No	35 (89.7)
Yes	4 (10.3)
Side of operation	
Right	23 (59)
Left	16 (41)
Extension of the surgery	
Isthmus excised	17 (43.6)
Isthmus not excised	22 (56.4)
Department	
Otolaryngology	21 (53.8)
General surgery	18 (46.2)

Seventeen (43.6%) patients developed biochemical hypothyroidism as they had a postoperative level of more than 4.4 mIU/L (Table 2 and Table 3). The majority of our patients (n = 11, 64.71%) developed hypothyroidism in the first six months postoperatively, three (17.64%) in the first 12 months, two (11.76%) in 18 months, and one (5.89%) in 24 months, as shown in Table 4.

**Table 2:** Preoperative TSH levels TSH: thyroid-stimulating hormone

TSH level (mIU/L)	Number of patients	Percentage (%)
0.2-0.49	3	7.7
0.5-2.39	32	82.1
2.4-4.4	4	10.3

**Table 3:** Postoperative TSH levels TSH: thyroid-stimulating hormone

TSH level (mIU/L)	Number of patients	Percentage (%)
0.2-0.49	1	2.6
0.5-2.39	12	30.8
2.4-4.4	9	23.1
>4.4	17	43.6

**Table 4:** Time taken to develop biochemical hypothyroidism postoperatively

Months	Number of patients	Percentage (%)	Cumulative percentage (%)
6	11	64.71	64.71
12	3	17.64	82.35
18	2	11.76	94.11
24	1	5.89	100
Total	17	100	

Hemithyroidectomy significantly increases postoperative TSH levels (p < 0.001). Moreover, patients who underwent isthmusectomy with lobectomy had a higher chance of developing hypothyroidism (p = 0.044). Additionally, patients under the general surgery department had a higher incidence of developing hypothyroidism postoperatively (p = 0.003). On the other hand, we found no significant difference in gender, BMI, and history of DM between the postoperative euthyroid and hypothyroid groups. Table 5 compares the euthyroid and hypothyroid groups.

**Table 5:** Comparison between euthyroid and hypothyroid groups in gender, BMI, DM, side of operation, isthmusectomy, and department BMI: body mass index, DM: diabetes mellitus

Factors	Euthyroid	Hypothyroid	p-value
Gender			
Male	4	4	0.992
Female	18	13	
BMI (kg/m^2^)			
<18.5	2	1	0.965
18.5-24.9	7	5	
25-29.9	6	5	
30-34.9	5	3	
35-39.9	1	1	
>40	1	2	
DM			
Diabetic	2	1	0.709
Nondiabetic	20	16	
Resected lobe			
Right	12	11	0.755
Left	10	6	
Isthmusectomy			
Yes	6	11	0.044
No	16	6	
Department			
Otolaryngology	17	4	0.003
General surgery	5	13	

## Discussion

In our study, 17 (43.59%) patients developed hypothyroidism after hemithyroidectomy, comparable with the percentages reported in other studies [4,8,12,13]. Hemithyroidectomy can result in the potentially disabling consequence of hypothyroidism. However, prophylactic administration of thyroxine should not be immediately started as it needs to be closely monitored to prevent the emergence of subclinical hyperthyroidism. Furthermore, the residual thyroid tissue may not have the ability to regain its normal function following surgery if thyroxine is used [12]. TSH has a half-life of about seven days, and it takes approximately four to five half-lives for it to be cleared from the body [13]. In order to accurately estimate the synthesis of thyroid hormones by the remaining thyroid tissue, it is preferable to start measuring TSH levels not earlier than 12 weeks post-surgery. Hence, we urge researchers to investigate this topic further to identify the most suitable time to perform a postoperative thyroid function test.

We monitored the patients’ TSH levels for two years post-surgery, and it was found that the majority (64.71%) of patients who developed hypothyroidism did so in the first six months after surgery. However, a previous study showed that 33% of the patients who developed postoperative hypothyroidism returned to euthyroid state within 5-28 months after surgery without treatment [12]. Thus, the period to rule out the development of hypothyroidism related to the procedure cannot be accurately defined.

Previous studies mentioned risk factors for developing hypothyroidism following hemithyroidectomy, such as thyroiditis, antithyroid antibodies, high preoperative TSH levels, and lymphocytic infiltration [4,5,7,8,12,14-16]. In our study population, we found no similar risk factors; however, ismusthectomy, in addition to lobectomy, was a predictive risk factor for developing hypothyroidism.

Our study found that the rate of postoperative hypothyroidism is significantly lower in operations done by otolaryngology (19.05%) compared to general surgery (72.22%). Similarly, a previous study showed reduced rates of vocal fold palsy in procedures performed by otolaryngology (4.70%) when compared to general surgery (8.20%) [16].

Limitations

During the conduction of this study, we faced several limitations, including the lack of some variables that would have been helpful to better understand the relationship between hemithyroidectomy and the occurrence of hypothyroidism, such as the weight of the resected thyroid tissue and histopathological diagnosis. Although most patients developed hypothyroidism within six months of surgery, it was impossible to pinpoint the exact time for developing hypothyroidism because of the retrospective nature of the study.

## Conclusions

Our findings show that hemithyroidectomy significantly increases postoperative TSH. The overall incidence of hypothyroidism within two years of hemithyroidectomy is 43.59%. Among those who developed hypothyroidism, the majority (64.71%) did so within the first six months. Thus, we strongly recommend continuous monitoring of TSH levels in the first six months postoperatively, as it may serve beneficial in early detection and guide the decision of starting treatment before symptoms develop.
